# The effects of structured aerobic exercise and mind–body exercise on cognitive function in older adults with MCI: Systematic review and meta-analysis

**DOI:** 10.1097/MD.0000000000047633

**Published:** 2026-03-06

**Authors:** Xiangbo Wang, Yingli Liu, Yuheng Yin, Hui Huang, Jie Chen, Ziyi Chen, Songlin Liu, Linxiao Xiao, Shan Chen, Chenggen Peng

**Affiliations:** a Sport Institute, Hunan Agricultural University, Changsha, China; b Department of Neurosurgery, Xiangya Hospital, Central South University, Changsha, China; f Department of Spinal Surgery, Xiangya Hospital, Central South University, Changsha, China.; c Sport Institute, Hunan Normal University, Changsha, China; d College of Physical Education and Health, Guangxi Normal University, Guangxi, China; e Physical Science College, Changsha Normal University, Changsha, China

**Keywords:** aerobic exercise, cognitive functioning, mild cognitive impairment, mind–body exercise

## Abstract

**Background:**

Global aging has increased the prevalence of dementia, with mild cognitive impairment (MCI) representing a critical window for intervention. While exercise is recognized for mitigating cognitive decline, the comparative effectiveness of mind–body versus structured aerobic exercise remains unclear.

**Methods:**

Search sources included PubMed, Web of Science, and the Cochrane Library. Randomized controlled trials (RCTs) assessing mind–body exercise (tai chi, yoga, and dance) or structured aerobic exercise (walking and cycling) in patients with MCI aged over 50 years were included. The Mini-Mental State Examination (MMSE), Montreal Cognitive Assessment (MoCA), and Alzheimer’s Disease Assessment Scale–Cognitive Subscale (ADAS-Cog) were used as outcome measures. Random- or fixed-effects meta-analyses were conducted using RevMan 5.4.1. Heterogeneity was assessed using the *I*^2^ statistic. Subgroup analyses examined intervention parameters.

**Results:**

Twenty-six randomized controlled trials (n = 2,555) were included. Mind–body exercise significantly improved MMSE (mean difference [MD] = 1.27, 95% confidence interval [CI]: 0.99–1.55, *P* < .01), MoCA (MD = 1.89, 95% CI: 0.78–3.00, *P* = .0008), and ADAS-Cog (MD = −2.09, 95% CI: −2.94 to −1.25, *P* < .00001) versus controls. Structured aerobic exercise showed non-significant effects on MMSE (MD = 0.37, *P* = .21) and MoCA (MD = −0.49, *P* = .26), with modest improvement on ADAS-Cog (MD = −1.41, *P* = .002). Optimal mind–body parameters include ≥20 weeks’ duration, ≥60 minutes per session, and ≥3 times per week.

**Conclusions:**

Mind–body exercise demonstrates superior cognitive benefits compared with structured aerobic exercise in older adults with MCI. It is advised to prioritize mind–body exercise interventions at least 3 times per week, for 60 minutes per session, for at least 20 weeks. Limitations include heterogeneity and geographic bias; these findings warrant confirmation through multicenter trials.

## 1. Introduction

### 1.1. Background and significance

The global population is experiencing accelerated aging, leading to a heightened disease burden among the elderly, with dementia being particularly prevalent. Dementia is a widespread condition, affecting approximately 55 million individuals worldwide, a figure anticipated to increase to 150 million by 2050.^[1]^ Mild cognitive impairment (MCI), an intermediate stage between normal aging and clinical dementia, typically emerges before a definitive dementia diagnosis. This condition exhibits an annual conversion rate of up to 10% to 15% for progressing to dementia,^[2]^ underscoring its significance as a transitional phase that necessitates effective intervention. Consequently, implementing effective interventions during this conversion window is essential to decelerate the progression of dementia. Evidence suggests that pharmacological interventions have limited efficacy in treating MCI, whereas exercise has demonstrated a greater capacity to mitigate the progression of dementia.^[3,4]^ As a result, current research increasingly focuses on exercise intervention strategies.

These interventions, designed to improve cognitive function, have attracted considerable attention due to their proven effectiveness. A substantial body of research has demonstrated that structured aerobic exercise enhances cerebral perfusion efficiency. This has led to greater recognition of exercise as a crucial strategy for combating cognitive decline. The mechanisms involve upregulation of vascular endothelial growth factor, improvements in hippocampal function, and enhanced cerebrovascular reactivity, collectively contributing to the mitigation of cognitive decline and the enhancement of cognitive function.^[5–9]^ Structured aerobic exercises are characterized by repetitive and regular movements, serving as their fundamental features. This form of exercise is characterized by continuous execution at low to moderate intensity, facilitated by cyclic movements involving 1 or more body parts, such as alternating limb movements. The hallmark of this exercise type is the regularity of movement, exemplified by activities like walking, running, swimming, cycling, and jumping rope. The movement trajectory and force generation exhibit cyclic repetition, necessitating the coordination of multiple muscle groups to maintain movement consistency.^[10]^ Structured aerobic exercises, such as walking and cycling, are recognized for their ability to enhance cerebrovascular blood flow and increase levels of brain-derived neurotrophic factors. However, current evidence suggests that the cognitive benefits of these exercises may be limited due to their reliance on repetitive movement patterns and minimal cognitive engagement, resulting in only modest improvements in cognitive function.^[11,12]^ While some studies have reported that walking interventions do not significantly enhance cognitive function in older adults with MCI,^[13]^ other research has demonstrated positive outcomes from treadmill walking in the management of dementia.^[14]^ Despite these findings, there remains considerable academic debate regarding the effectiveness of structured aerobic exercises (including walking, cycling, and running)in enhancing cognitive performance among individuals with MCI. This underscores the necessity for further research to clarify optimal intervention designs.

Previous research indicates that complex, multimodal mind–body exercises exert a more pronounced impact on memory, cognitive function, and executive function in older adults than single, structured aerobic exercises.^[15,16]^ Previous meta-analytic studies have predominantly focused on specific types of physical activity, with limited comparative research exploring the effects of mind–body exercises versus structured aerobic exercise on cognitive function in older adults with MCI. Furthermore, the existing literature has primarily concentrated on exercise intervention trials and narrative reviews, with less emphasis on the detailed optimization of intervention protocols. This gap has led to a lack of practical guidance for designing daily exercise programs tailored to enhance cognitive function in this population. To address these limitations, the present study conducts a systematic review to analyze and compare the effects of structured aerobic exercises and mind–body exercise on cognitive outcomes in older adults with MCI. By investigating the unique benefits of mind–body practices in enhancing cognitive function, this study aims to inform the optimization of exercise-based intervention strategies for this vulnerable demographic.

## 2. Methods

### 2.1. Literature search strategy

A comprehensive search of multiple authoritative databases was conducted, including PubMed, Web of Science, and the Cochrane Library, covering the period from the inception of each database until April 2025. The search strategy employed both subject terms and free-text terms, encompassing keywords related to exercise (such as aerobic exercise, mind–body exercise, and physical activity), cognitive impairment (including MCI and dementia), older adults (such as elderly and senior individuals), and randomized controlled trials (RCTs). The retrieved literature was managed using EndNote X21, which facilitated the organization and removal of duplicate entries. Initial screening involved reviewing the titles and abstracts, followed by a full-text review to exclude irrelevant studies. Reasons for exclusion and the number of excluded studies were documented to determine the final selection of studies for inclusion. Two researchers independently screened, extracted, and cross-checked the data. Any disagreements were resolved through discussion, with consultation from a 3rd researcher if necessary. This meta-analysis was prospectively registered in PROSPERO (CRD 420251028638).

### 2.2. Inclusion and exclusion criteria

The study design specified for inclusion was an RCT, with the study population comprising elderly individuals diagnosed with MCI, without age or gender restrictions. The interventions included in the trial group were mind–body exercises, such as Tai Chi, dance, yoga, baduanjin, and other exercises emphasizing the integration of mind and body, as well as structured aerobic exercises, such as running, brisk walking, and bicycling. The control group received routine nursing care, balance stretching training, health education, or no exercise intervention. Cognitive function was assessed using the Mini-Mental State Examination (MMSE), the Montreal Cognitive Assessment (MoCA), and the Alzheimer Disease Assessment Scale-Cognitive Subscale (ADAS-Cog) as outcome measures.

Studies were excluded from the analysis if they did not utilize RCT designs, were published in non-English languages, described unclear or non-compliant intervention protocols, lacked accessible outcome data, or included participants with comorbidities that severely impacted cognitive function. A standardized table was developed to document the baseline characteristics of the included studies. Literature screening and data extraction were conducted independently by 2 researchers, who collected information on study demographics (authors, publication year, country), participant characteristics (sample size, age), intervention details (exercise type, frequency, intensity, duration), and primary outcome measures, including the MMSE, MOCA, and ADAS-Cog. Any discrepancies encountered during data extraction were resolved through collaborative discussion or consultation with a third expert to ensure accuracy and consistency.

### 2.3. Search strategy of PubMed

(1) (((((((((((exercise[MeSH Terms]) OR (physical exercises[Title/Abstract])) OR (aerobic exercise[Title/Abstract])) OR (aerobic dance[Title/Abstract])) OR (walking[Title/Abstract])) OR (running[Title/Abstract])) OR (bicycle riding[Title/Abstract])) OR (mind body exercise[Title/Abstract])) OR (tai chi[Title/Abstract])) OR (tai ji[Title/Abstract])) OR (baduanjin[Title/Abstract])) OR (mindful exercise[Title/Abstract]).
(2) (((((((((mild cognitive impairment[MeSH Terms]) OR (MCI[Title/Abstract])) OR (dementia[Title/Abstract])) OR (memory disorder[Title/Abstract])) OR (cognitive decline[Title/Abstract])) OR (memory impairment[Title/Abstract])) OR (old people[Title/Abstract])) OR (elderly[Title/Abstract])) OR (aged[Title/Abstract])) OR (aging[Title/Abstract]).
(3) (((randomized controlled trial[Publication Type]) OR (controlled clinical trial[Publication Type])) OR (randomized[Title/Abstract])) (((((((((((((exercise[MeSH Terms]) OR (physical exercises[Title/Abstract])) OR (aerobic exercise[Title/Abstract])) OR (aerobic dance[Title/Abstract])) OR (walking[Title/Abstract])) OR (running[Title/Abstract])) OR (bicycle riding[Title/Abstract])) OR (mind body exercise[Title/Abstract])) OR (tai chi[Title/Abstract])) OR (tai ji[Title/Abstract])) OR (baduanjin[Title/Abstract])) OR (mindful exercise[Title/Abstract])) AND ((((((((((mild cognitive impairment[MeSH Terms]) OR (MCI[Title/Abstract])) OR (dementia[Title/Abstract])) OR (memory disorder[Title/Abstract])) OR (cognitive decline[Title/Abstract])) OR (memory impairment[Title/Abstract])) OR (old people[Title/Abstract])) OR (elderly[Title/Abstract])) OR (aged[Title/Abstract])) OR (aging[Title/Abstract]))) AND (((randomized controlled trial[Publication Type]) OR (controlled clinical trial[Publication Type])) OR (randomized[Title/Abstract])).

### 2.4. Data analysis plan

In this study, continuous outcome indicators were analyzed using RevMan 5.4.1 (Cochrane, London, United Kingdom), with the mean difference (MD) and 95% confidence interval (CI) as the effect size metrics. Data synthesis was conducted using either a fixed-effects or random-effects model, selected based on the following prespecified criteria: a random-effects model was applied when substantial heterogeneity was detected (*I*^2^ ≥ 50%); otherwise, a fixed-effects model was used. The magnitude of heterogeneity was assessed using the *I*^2^ statistic, and heterogeneity was categorized as low (*I*^2^ ≤ 25%), moderate (25% < *I*^2^ ≤ 50%), or high (*I*^2^ > 50%). This methodological approach ensured appropriate data pooling while accounting for inter-study variability in effect estimates. Analyses were performed to explore sources of heterogeneity. Formal interaction tests were conducted using RevMan subgroup comparison function, which employs a chi-square test to determine whether effect sizes differ significantly between subgroups (*P*-interaction < .05 indicates a statistically significant difference).

## 3. Results

### 3.1. Literature search and study selection

**Figure 1.**
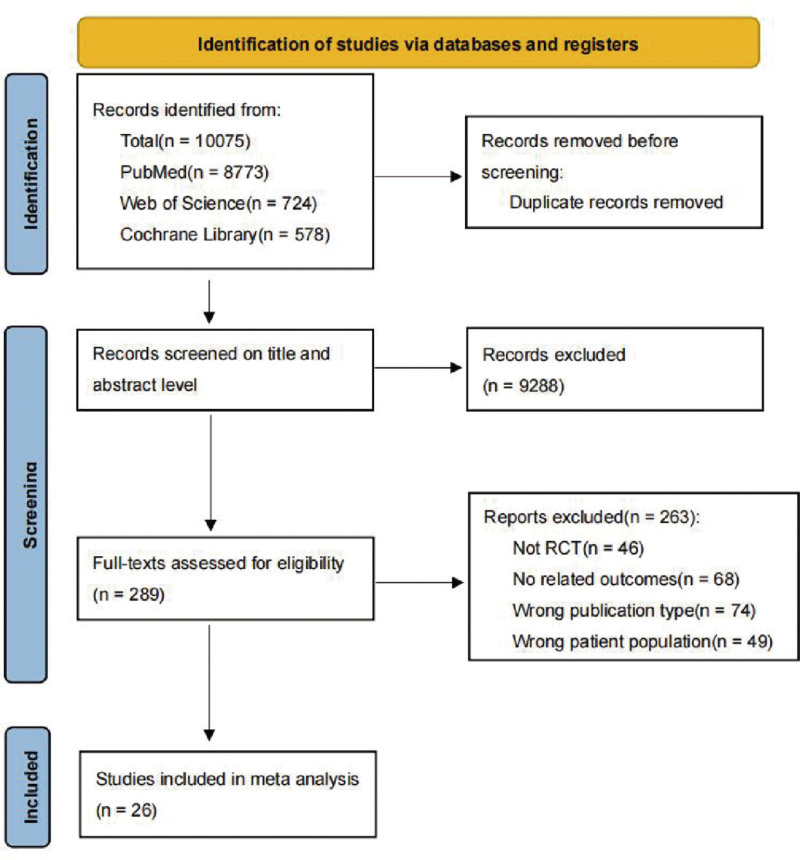
Literature retrieval flowchart.

An extensive database search initially yielded 10,075 pertinent documents. After eliminating 498 duplicate records, 9288 studies were excluded through title and abstract screening, leaving 289 articles selected for full-text evaluation. Of these, 263 did not satisfy the predefined inclusion criteria: 36 were non-RCT, 68 lacked relevant outcome measures, 84 were of inappropriate publication types, 49 involved incorrect patient populations, and 8 were non-English publications. Following this rigorous screening process, 26 articles were deemed eligible for inclusion in the present study, thereby ensuring compliance with systematic review standards and a focus on pertinent evidence (Fig. 1).

### 3.2. Literature characteristics

Twenty-six studies were included in the final analysis, involving 2555 participants. Of these, 13 studies focused on structured aerobic exercises,^[14,17–28]^ comprising 542 individuals in the experimental group and 515 in the control group. Fourteen studies examined mind–body exercise interventions,^[21,29–41]^ with 709 participants allocated to the experimental group and 789 to the control group. This distribution allowed for a comparative analysis of the 2 exercise modalities across relevant outcomes in older adults with MCI. Studies were from China (n = 11) ^[21,23,30,31,34,35,37–41]^, America (n = 4),^[14,19,29,32]^ Canada (n = 4),^[18,26–28]^ Australia (n = 1),^[25]^ Denmark (n = 1),^[20]^ Japan (n = 1),^[36]^ Slovakia (n = 1),^[17]^ Spain (n = 1),^[22]^ Netherlands (n = 1), and^[24]^ Greece (n = 1).^[33,42]^ Subjects maintained their original medication use and dietary habits. Structured aerobic and mind–body exercise were included in the study. The control group underwent balanced stretching exercises, health education, or no exercise intervention (Table 1).

**Table 1:** Characteristics of the included studies.

Structured aerobic exercise						
Study	Country	Years	Participants	Exercise intervention	Control	Outcomes
Ambrose et al^[28]^	Canada	Average age of 74	Experimental = 35 Control = 35	3 times a week, 60 min each time, 24 wk, 40–70% HRR, walking	No exercise intervention	ADAS-Cog
Arcoverde et al^[14]^	America	Average age of 78.5	Experimental = 20 Control = 10	Twice a week, 30 min each time, 4 mo, 40–60% HRR, treadmill, stationary bike or elliptical machine	Maintain clinical and drug treatment	MMSE
Davis et al^[26]^	Canada	70–80 yr old	Experimental = 30 Control = 28	Twice a week, 60 min each time, 5 mo, 60% HRR, outdoor walking	Balance stretching training	MOCA
Hagovska (2016)	Slovakia	Average age of 67.07 ± 4.3	Experimental = 40 Control = 40	7 times a week, 30 min each time, 10 wk, various forms of walking and climbing stairs	No exercise intervention	MMSE
Hoffmann (2015)	Denmark	50–90 yr old	Experimental = 102 Control = 88	3 times a week, 60 min each time, 16 wk, moderate intensity, cycling, cross training bike and treadmill	No exercise intervention	MMSE; ADAS-Cog
Hsu (2017)	Canada	Average age of 73.03	Experimental = 10 Control = 11	Twice a week, 30 min each time, 6 mo, 60–70% HRR, walking	No exercise intervention	MMSE; MOCA
Lautenschlage et al^[25]^	Australia	50 yr old and above	Experimental = 85 Control = 85	3 times a week, 50 min each time, 24 wk, walking	Health course education	ADAS-Cog
Nagsmatsu (2013)	Canada	70–80 yr old	Experimental = 30 Control = 26	Twice a week, 60 min each time, 28 wk, 40–80% HRR, walking	Health course education	MMSE; MOCA
Song and Yu^[23]^	China	60 yr old and above	Experimental = 60 Control = 60	3 times a week, 60 min each time, 16 wk, walking	Health education	MOCA
Tao et al^[21]^	China	60 yr old and above	Experimental = 20 Control = 20	3 times a week, 60 min each time, 24 wk, 55–75% HRR, walking and Ba duan jin	No exercise intervention	MOCA
Tomoto et al^[19]^	America	55–80 yr old	Experimental = 22 Control = 30	3 or 3–4 times a week, 25–30 or 30–35 min each time, 12 mo, walking	Stretching exercise	MMSE
Van Uffelen et al^[24]^	Netherlands	70–80 yr old	Experimental = 71 Control = 67	Twice a week, 60 min each time, 12 mo, moderate intensity, walking	Relaxation and Balance Training	MMSE
Varela (2011)	Spain	60 yr old and above	Experimental = 17 Control = 15	3 times a week, 30 min each time, 3 mo, 40% HRR and 60% HRR, Bicycle	No exercise intervention	MMSE

Mind–body exercise						
Study	Country	Years	Participants	Exercise Intervention	Control	Outcomes
Chan et al^[30]^	China	60 yr old and above	Experimental = 27 Control = 25	Twice a week, 60 min each time, 2 mo, Tai Chi	Health education	MMSE
Chang et al^[38]^	China	60 yr old and above	Experimental = 62 Control = 47	3 times a wk, 30 min each time, 18 wk, square dance	No exercise intervention	MOCA
Doi et al^[36]^	Japan	Average age of 76	Experimental = 67 Control = 67	Once a week, 60 min each time, 40 wk, aerobic dance	Health education	MMSE
Lam et al^[41]^	China	Average age of 65	Experimental = 92 Control = 169	At least 3 times a week, 30 min or more each time, 12 mo, Tai Chi	Stretching or conditioning exercise	MMSE; ADAS-Cog
Lazarou et al^[33]^	Greece	Average age of 66.8 ± 10	Experimental = 63 Control = 66	Twice a week, 60 min each time, 10 mo, international ballroom dance	No exercise intervention	MMSE; MOCA
Li et al^[27]^	America	65 yr old and above	Experimental = 22 Control = 23	Twice a week, 60 min each time, 14 wk, Tai Chi	No exercise intervention	MMSE
Li et al^[40]^	China	Average age of 74.6	Experimental = 23 Control = 22	Twice a week, 60 min each time, 16 wk, Tai Chi	No exercise intervention	MOCA
Linda (2010)	China	60 yr old and above	Experimental = 171 Control = 218	More than 3 times a wk, 30 min or more each time, 12 mo, Tai Chi	Stretching exercise	MMSE; ADAS-cog
Ming (2019)	China	50–85 yr old	Experimental = 19 Control = 19	3 times a week, 35 min each time, 3 mo, 60–80% HRR, aerobic dance	No exercise intervention	MMSE; MOCA
Siu et al^[35]^	China	60 yr old and above	Experimental = 80 Control = 80	Twice a week, 60 min each time, 16 wk, Tai Chi	No exercise intervention	MMSE
Tao et al^[21]^	China	60 yr old and above	Experimental = 20 Control = 20	3 times a week, 60 min each time, 24 wk, 55–75% HRR, Ba duan jin	No exercise intervention	MOCA
Tsai et al^[32]^	America	60 yr old and above	Experimental = 28 Control = 27	3 times a week, 40 min each time, 20 wk, Tai Chi	No exercise intervention	MMSE
Xu et al^[31]^	China	60–80 yr old	Experimental = 6 Control = 6	3 times a week, 30 min or more each time, 12 wk, Tai Chi	No exercise intervention	MOCA; ADAS-Cog
Zhu et al^[37]^	China	50–85 yr old	Experimental = 29 Control = 31	3 times a week, 35 min, 3 mo, 60–80% HRR, aerobic dance	No exercise intervention	MOCA

ADAS-Cog = Alzheimer Disease Assessment Scale-cognitive, HRR = Heart Rate Reserve; MMSE = Mini-Mental State Examination, MOCA = Montreal Cognitive Assessment.

### 3.3. Quality of studies and risk of bias

The Cochrane Risk of Bias Assessment Tool,^[42]^ was employed to assess potential biases in the included studies across several domains: random sequence generation, allocation concealment, blinding, data completeness, selective reporting, and other sources of bias. Studies were categorized as low-, medium-, or high-risk based on predefined criteria. Two independent reviewers conducted the assessments, and any disagreements were resolved through discussion or consultation with a 3rd expert to ensure consistency.

**Figure 2.**
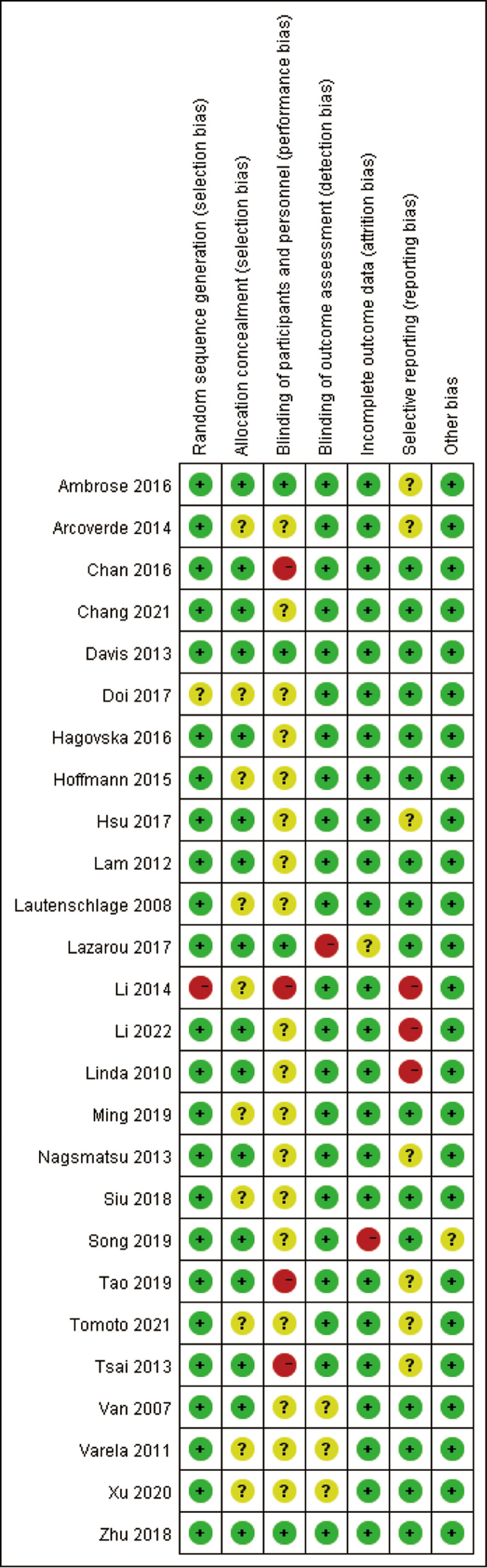
Risk of bias summary of review authors judgements about each risk of bias item for each included study.

The findings indicated that 25 studies had a low risk of random sequence generation, while 16 studies had a low risk of allocation concealment. 4 studies achieved low risk of blinding of participants and personnel, and 22 studies demonstrated low risk of blinding in outcome assessment. Furthermore, 24 studies were identified as having a low risk of incomplete outcome data, 16 studies were considered to have a low risk for selective reporting, and 25 studies showed a low risk of other biases. Overall, the included literature demonstrated a low risk of bias, characterized by high methodological quality and representativeness. This rigorous assessment underscores the reliability of the included studies for synthesizing evidence on exercise interventions in MCI (Fig. 2).

### 3.4. Publication bias

To evaluate potential publication bias, we conducted Egger linear regression test on all included studies. The analysis revealed an intercept of 1.93 (SE = 0.96, 95% CI: −0.04 to 3.89, *P* = .054). Although this *P*-value slightly exceeds the conventional significance threshold (*P* < .05), it approaches borderline significance, suggesting a possible trend toward small-study effects or publication bias. Nevertheless, the funnel plot demonstrated approximate symmetry (Figures S1 and S2, Supplemental Digital Content, https://links.lww.com/MD/R347), with no evidence of missing small-sample studies in the lower quadrants. Collectively, these results indicate a low risk of publication bias, though its potential influence cannot be entirely excluded.

### 3.5. Meta-analysis results: cognitive outcome comparisons

This meta-analysis assessed the effects of structured aerobic and mind–body exercise on cognitive function-related metrics, including the MMSE, MOCA, and ADAS-Cog. These widely utilized instruments were chosen to provide a comprehensive evaluation of global cognitive status, specific cognitive domains, and disease-specific cognitive decline in older adults with MCI. This approach facilitates a nuanced comparison of the effects of the 2 exercise modalities across various cognitive dimensions.

#### 3.5.1. MMSE findings

**Figure 3.**
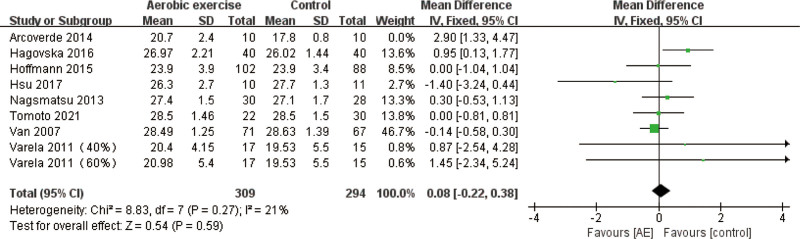
Sensitivity analysis of effect sizes of forest plots for structured aerobic exercise versus controls – MMSE outcomes. MMSE = Mini-Mental State Examination.

For the MMSE outcome, 8 studies,^[14,17–20,22,24,27]^ involving 623 participants (319 in the experimental group, 304 in the control group) were included in the analysis of structured aerobic exercises. The pooled effect size comparing structured aerobic exercise with the control group was MD = 0.37 (95% CI: 0.21–0.96, *P* = .21), indicating no statistically significant difference. Heterogeneity assessment revealed high inter-study variability (*I*^2^ = 62%, *P* = .008), prompting the use of a random-effects model for data synthesis (Figure S3, Supplemental Digital Content, https://links.lww.com/MD/R347). Sensitivity analysis was conducted to identify sources of heterogeneity, revealing that excluding the Arcoverde 2014 study,^[14]^ the statistics showed robust results (*I*^2^ = 21%, [*P* = .59]) (Fig. 3), and reading the full text, it was found that there were both Alzheimer disease and mixed dementia patients in the present study, and that the degree of dementia was limited only to a Clinical Dementia Rating Scale score of 1. Whereas the other study subjects in this group were MCI. It is possible that the differences in the study subjects led to the differences in this study, which in turn increased the heterogeneity.

Nine studies,^[29,30,32–36,39,41]^ examining mind–body exercise, were included, involving 1258 participants (569 in the experimental group and 689 in the control group). Pooled analysis revealed a significant effect size for mind–body exercise compared to controls, with an MD of 1.27 (95% CI: 0.99–1.55, *P* < .01). Heterogeneity testing indicated moderate inter-study variability (*I*^2^ = 55%, *P* = .02), justifying the use of a fixed-effects model for data synthesis (Figure S4, Supplemental Digital Content, https://links.lww.com/MD/R347).

**Figure 4.**
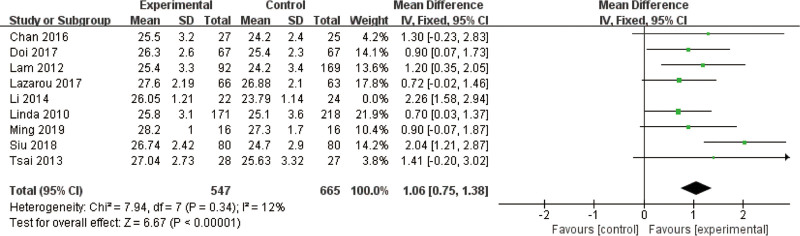
Sensitivity analysis of effect sizes of forest plots for mind–body exercise versus controls – MMSE outcome. MMSE = Mini-Mental State Examination.

Sensitivity analysis was conducted to address heterogeneity, revealing robust results (*P* = .34, *I*^2^ = 12%) after excluding the Li study^[27]^ (Fig. 4).

A detailed review of this study highlighted methodological discrepancies: it lacked proper randomization and blinding, and its intervention incorporated nontraditional Tai Chi with additional variables, deviating from the standardized mind–body practices (e.g., traditional Tai Chi, qigong) used in other included studies. These differences in study design (specifically regarding grouping methods, blinding procedures, and intervention fidelity) were identified as potential sources of heterogeneity, underscoring the importance of consistent methodological reporting in future trials. This analysis reinforces the robustness of mind–body exercise effects when studies adhere to standardized intervention protocols and rigorous trial design.

#### 3.5.2. MOCA findings

**Figure 5.**
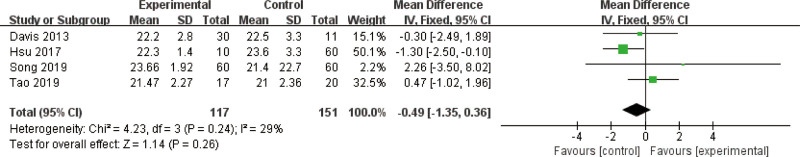
Effect sizes of forest plots for structured aerobic exercise versus controls – MOCA outcomes. MoCA = Montreal Cognitive Assessment.

For the MOCA outcome, 4 studies^[21,23,26,27]^ involving 268 participants were included in the analysis of structured aerobic exercise. The pooled MD comparing structured aerobic exercises to the control group was −0.49 (95% CI: −1.35 to 0.36, *P* = .26), indicating no statistically significant effect. Heterogeneity assessment revealed low inter-study variability (*I*^2^ = 29%, *P* = .24), confirming consistent results across studies for the impact of structured aerobic exercises on MOCA scores (Fig. 5).

**Figure 6.**
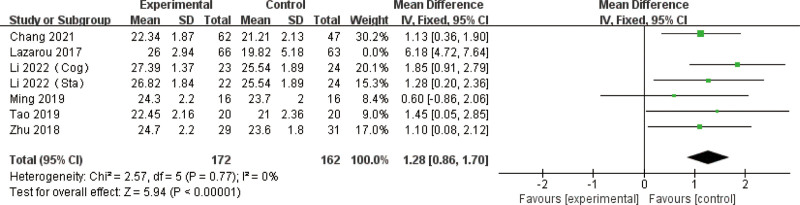
Sensitivity analysis of effect sizes of forest plots for mind–body exercise versus controls – MOCA outcomes. MoCA = Montreal Cognitive Assessment.

In the analysis of mind–body exercises, 6 studies^[21,33,37–40]^ encompassing 268 participants (117 in the experimental group and 151 in the control group) were included. The aggregated effect size demonstrated a significant positive impact of mind–body exercises compared to the control groups, with an MD of 1.89 (95% CI: 0.78–3.00, *P* = .0008). However, tests for heterogeneity revealed substantial inter-study variability (*I*^2^ = 86%, *P* < .01), indicating considerable differences in effect estimates despite the overall significant improvement in MOCA scores (Figure S5, Supplemental Digital Content, https://links.lww.com/MD/R347). Sensitivity analysis was conducted to further investigate the source of heterogeneity, and the results were found to be robust (*P* < .01, *I*^2^ = 0%) upon excluding Lazarou 2017^[12]^ (Fig. 6).

Upon reviewing the full text and comparing it with other studies within the same group, it was identified that the intervention in this study involved international ballroom dancing, whereas the other studies employed taijiquan, Baduanjin, and aerobic dance. The complex, intricate movements associated with international social dance may contribute to variations in intervention effects, thereby increasing heterogeneity.

#### 3.5.3. ADAS-Cog findings

**Figure 7.**
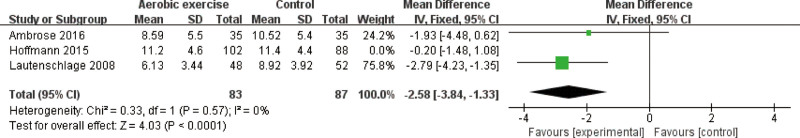
Sensitivity analysis of effect sizes of forest plots for structured aerobic exercise versus controls – ADAS-Cog outcomes. ADAS-Cog = Alzheimer Disease Assessment Scale-Cognitive Subscale.

Regarding the ADAS-Cog index, 3 studies,^[20,25,28]^ were incorporated into the analysis of structured aerobic exercises, involving a total of 360 participants. Among them, 185 were in the experimental group and 175 were in the control group. The pooled effect size for the structured aerobic exercise group compared to the control group was MD (95% CI = −1.41 (−2.31 to −0.52), *P* = .002). The heterogeneity test results showed high heterogeneity (*I*^2^ = 72%, *P* = .03) (Figure S6, Supplemental Digital Content, https://links.lww.com/MD/R347). To further explore the source of this heterogeneity, a sensitivity analysis was carried out. It was found that after excluding the Hoffmann 2015 study,^[18]^ the statistical results became more robust (*P* < .01, *I*^2^ = 0%) (Fig. 7).

By carefully examining this study and comparing it with other studies in the same group, it was discovered that this study included Alzheimer disease patients, while the other studies focused on MCI subjects. This difference might be the cause of the observed heterogeneity.

**Figure 8.**
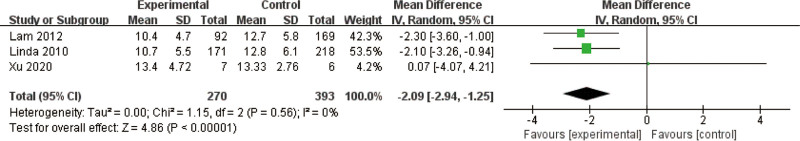
Effect sizes of forest plots for mind–body exercise versus controls – ADAS-Cog outcomes. ADAS-Cog = Alzheimer Disease Assessment Scalee-Cognitive Subscale.

For mind–body exercise, 3 studies ^[31,34,41]^with a total of 663 participants (270 in the experimental group and 393 in the control group) were included. The combined effect size of the mind–body exercise group and the control group was MD (95% CI = −2.09 (−2.94 to −1.25), *P* < .00001), and there was no heterogeneity (*I*^2^ = 0%, *P* = .56). This indicates that mind–body exercise is effective in reducing ADAS-Cog scores (Fig. 8).

### 3.6. Subgroup analyses: effects of intervention parameters on cognitive outcomes

Subgroup analyses were performed to assess the effects of various parameters of mind–body exercise interventions (specifically, duration, session length, and frequency) on MMSE scores, encompassing a total sample of 2067 participants. Among the 6 predefined subgroups, 3 demonstrated high heterogeneity, while the other 3 exhibited no significant inter-study variability. This suggests that these parameters have differential impacts on cognitive outcomes in older adults with MCI. Notable discrepancies in intervention effect sizes across different subgroup combinations were observed for MMSE scores, particularly within subgroups characterized by high heterogeneity. This indicates that variations in the design of exercise protocols contributed to these differences. Mind–body exercise demonstrated a significant positive effect compared with control groups, with a pooled MD of 1.27 (95% CI: 1.11–1.43, *P* < .01), highlighting its overall efficacy in improving global cognitive status. These findings underscore the importance of tailoring intervention protocols to specific parameters, such as weekly frequency and session duration, to optimize cognitive benefits while minimizing methodological heterogeneity in future trials.

**Figure 9.**
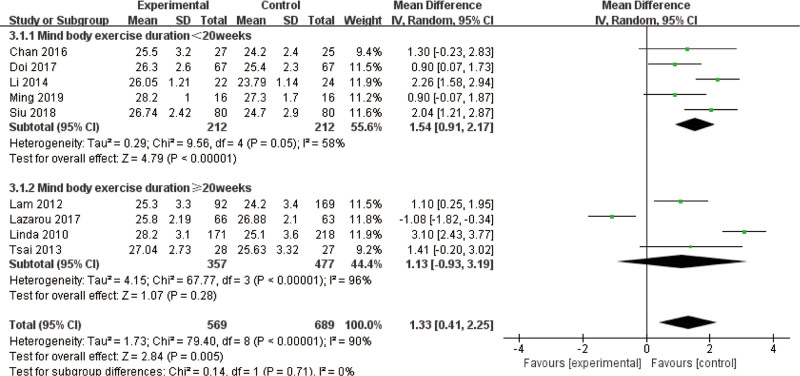
Subgroup analysis of mind–body exercise MMSE outcomes. MMSE = Mini-Mental State Examination (duration).

Both subgroups, categorized by mind–body exercise duration of <20 weeks and 20 weeks or more, demonstrated the superiority of the mind–body exercise group over the control group. The combined effect sizes for the subgroup with a duration of <20 weeks were (MD = 1.63, 95% CI = 1.24–2.02, *P* < .01), while for the subgroup with a duration of 20 weeks or more, the effect sizes were (MD = 0.87, 95% CI = 0.46–1.29, *P* < .01). However, the subgroup with a duration of <20 weeks exhibited moderate heterogeneity (*I*^2^ = 58%), whereas the subgroup with a duration of 20 weeks or more showed no heterogeneity (*I*^2^ = 0%). But statistical testing (*P* = .71) showed that this difference is likely due to chance and cannot prove that short-term interventions are more effective (Fig. 9).

**Figure 10.**
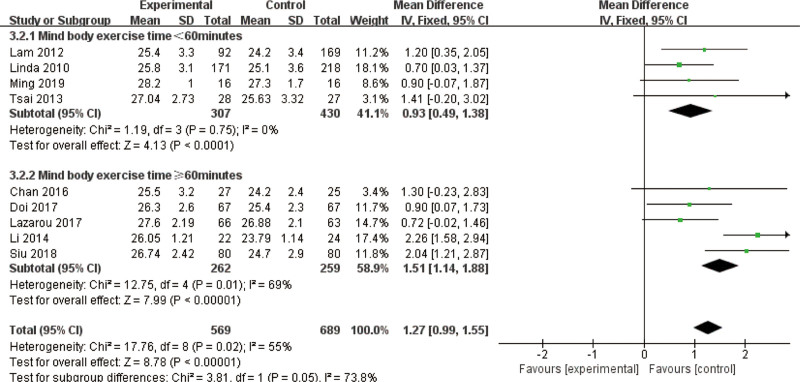
Subgroup analysis of mind–body exercise MMSE outcomes. MMSE = Mini-Mental State Examination (frequency).

Subgroups categorized by weekly exercise frequency (fewer than 3 sessions versus 3 or more) consistently demonstrated the superiority of mind–body exercise across all cognitive measures. While mind–body interventions positively impacted MMSE scores regardless of session duration or weekly frequency, longer sessions appeared to confer greater cognitive benefits despite moderate inter-study variability. Notably, shorter sessions exhibited no significant heterogeneity, underscoring the reliability of frequent, shorter mind–body exercise programs for older adults with MCI (Fig. 10).

**Figure 11.**
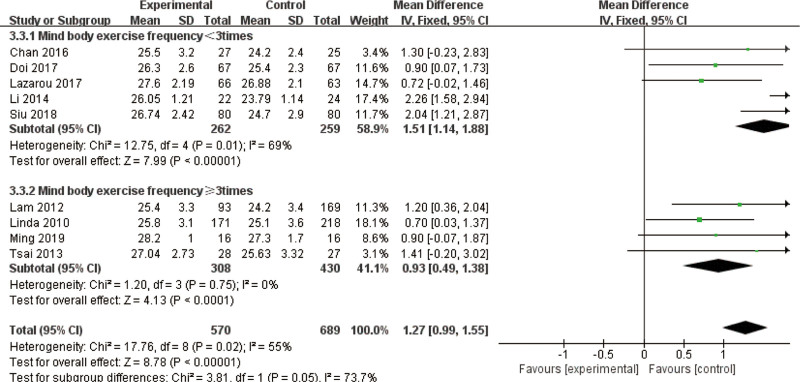
Subgroup analysis of mind–body exercise MMSE outcomes. MMSE = Mini-Mental State Examination (time).

When further categorized by session length (<60 minutes vs 60 minutes or more) both subgroups demonstrated significant benefits for the mind–body exercise group. The pooled analysis revealed an MD of 0.93 (95% CI: 0.49–1.38, *P* < .01) for sessions lasting <60 minutes, indicating moderate improvements. Conversely, sessions of 60 minutes or longer demonstrated a more substantial MD of 1.51 (95% CI: 1.14–1.88, *P* < .01). The subgroup with longer-duration sessions exhibited moderate inter-study variability (*I*^2^ = 69%), whereas the subgroup with shorter-duration sessions exhibited no significant heterogeneity (*I*^2^ = 0%), highlighting the consistency of results for shorter sessions. The subgroup difference test (*x*^2^ = 3.81, df = 1, *P* = .05) indicated that mind–body exercise sessions ≥ 60 minutes significantly outperformed shorter sessions in improving MMSE scores (Fig. 11).

The analysis of pooled effect sizes indicated an MD of 1.51 (95% CI: 1.14–1.88, *P* < .01) for the subgroup engaging in exercise less than 3 times per week, which was associated with moderate heterogeneity (*I*^2^ = 69%). In contrast, the subgroup participating in exercise 3 or more times per week exhibited an MD of 0.93 (95% CI: 0.49–1.38, *P* < .01) and demonstrated no heterogeneity (*I*^2^ = 0%). The subgroup difference test (*x*^2^ = 3.81, df = 1, *P* = .05) concluded that mind–body exercise performed < 3 times weekly demonstrated significantly greater improvement in MMSE scores than exercise performed ≥ 3 times per week.

## 4. Discussion

This study employed a systematic meta-analysis to compare the impacts of mind–body exercise and structured aerobic exercise on cognitive function in older adults with MCI. Results indicated that mind–body exercise demonstrated significantly larger effect sizes compared to structured aerobic exercise across multiple cognitive (metrics: MMSE, MD = 1.27, *P* < .0002 and MOCA, MD = 1.89, *P* = .0008), in contrast to non-significant effects for structured aerobic exercise (MMSE: MD = 0.37, *P* = .21; MOCA: MD = −0.49, *P* = .26). Mind–body exercise yielded greater improvements in ADAS-Cog scores.

This discrepancy implies that the intervention mechanisms of mind–body exercise may extend beyond simple physiological stimulation, instead integrating multidimensional neurocognitive regulatory pathways. These findings highlight the potential of mind–body practices to engage complex cognitive–motor interactions, yielding more pronounced cognitive benefits than repetitive, structured aerobic activities. The enhanced effect sizes observed in mind–body exercise underscore its unique role in addressing cognitive decline in MCI, warranting further exploration of its neurobiological mechanisms and clinical applications.

Accumulating evidence indicates that the mechanisms through which exercise influences cognitive function are multidimensional, with structured aerobic exercises (characterized by repetitive movements without substantial cognitive engagement) potentially exerting limited effects on individual cognitive domains. Not all aerobic exercise modalities offer equivalent benefits for cognitive enhancement.^[43,44]^ A separate investigation examining the immediate effects of acute aerobic exercise on cognitive function in healthy older adults reported that, while such exercise can boost cognitive performance, the variability in study designs and the restricted scope of cognitive domains assessed suggest differential benefits across aerobic protocols.^[45]^ These findings highlight the importance of cognitive engagement within exercise interventions, particularly for older adults, and underscore the need to differentiate between exercise types when evaluating their cognitive benefits.

While aerobic exercise holds promise for cognitive enhancement, its efficacy is contingent on the degree of cognitive engagement and participant enjoyment.^[46]^ Thus, integrating components that amplify these factors may be essential to optimizing its cognitive benefits. In contrast, mind–body practices (such as Tai Chi and yoga) demonstrate substantial effects on improving cognitive function in older adults, primarily due to their unique integration of motor, cognitive, and emotional interventions. A pivotal differentiating factor lies in dual-task training, a core mechanism that distinguishes mind–body exercises from repetitive aerobic activities. This approach not only enhances physical health but also positively impacts neural processes by engaging higher-order cognitive functions.

Specifically, Tai Chi practice necessitates coordination of slow, deliberate movements with controlled breathing and mental focus, effectively challenging both motor and cognitive systems simultaneously. This dual-task demand (merging physical execution with attentional regulation) creates a neurocognitive environment that promotes synaptic plasticity and enhances network connectivity, mechanisms less emphasized in traditional aerobic routines. By engaging these integrated pathways, mind–body exercises may offer more comprehensive cognitive benefits, particularly in populations with MCI, where multifaceted interventions are increasingly recognized as critical for addressing cognitive decline. These distinctions highlight the importance of exercise design that incorporates cognitive–motor interactions, underscoring the need for tailored approaches to maximize therapeutic outcomes in aging populations.^[47]^ It not only improves physical health but also positively affects brain function. Tai Chi practice requires coordination of slow movements, breath regulation, and spatial orientation, and this complex task activates the frontoparietal network, which enhances working memory and executive function.^[48]^ Functional magnetic resonance imaging investigations have revealed that a 12-week Tai Chi intervention substantially strengthened functional connectivity between the default mode network (DMN) and dorsal attention network in individuals with MCI. This finding aligns with prior research indicating that mind–body practices such as Tai Chi can modulate the DMN via distinct neural pathways, potentially offering innovative approaches to prevent age-related conditions linked to DMN dysfunction.^[49]^

Extensive research has underscored the role of mind–body exercise in enhancing cognitive function via stress-regulatory mechanisms. For example, yoga (a prototypical mind–body practice) effectively reduces cortisol levels through its focus on deep breathing and meditation, thereby inhibiting hyperactivation of the hypothalamic–pituitary–adrenal axis. This regulatory process not only mitigates neuroinflammation but also diminishes oxidative stress damage, indirectly promoting cognitive improvements by safeguarding neural health.^[50–52]^ In contrast, while structured aerobic exercises such as brisk walking excel at enhancing cerebral hemodynamics (e.g., by increasing hippocampal blood flow) their ability to actively engage higher-order cognitive processes remains comparatively limited. These activities rely on repetitive motor patterns that fail to activate synergistic networks across multiple brain regions fully. However, emerging evidence suggests that integrating aerobic exercise with cognitive stimulation or adopting multimodal intervention strategies may amplify its cognitive benefits.^[53]^ This highlights the need for exercise protocols that balance physiological advantages with neurocognitive engagement, particularly for populations at risk of cognitive decline.

For older adults with MCI, mind–body exercise should be prioritized when designing exercise intervention programs, as it demonstrates greater efficacy than conventional exercise in improving global cognitive function and cognitive flexibility.^[54]^ Subgroup analysis results suggest that a weekly protocol of 3 60-minute mind–body exercise sessions is optimal. This recommendation not only aligns with the findings of this meta-analysis but also acknowledges the dual benefits of mind–body practices: enhancing cognitive function while simultaneously improving balance, coordination, and reducing fall risk, thereby promoting overall physical well-being in older populations.

For individuals with lower exercise tolerance, modifications to intensity and duration are essential. Personalized adjustments, such as reducing session length or intensity, can help ensure accessibility and safety without compromising potential cognitive benefits. When tailoring these programs, clinicians should also consider patients’ comorbid health conditions, personal preferences, and lifestyle factors to enhance adherence: critical for maximizing the effectiveness of exercise interventions. By integrating evidence-based parameters with individual needs, these customized mind–body exercise protocols can serve as a robust strategy to address cognitive decline while supporting holistic health in older adults with MCI.

Multimodal imaging techniques such as diffusion tensor imaging and functional near-infrared spectroscopy have been widely used in dementia research and have been applied to identify pathological changes in early dementia and to assess disease progression. Diffusion tensor imaging can be used to assess the integrity of white matter fiber tracts in the brain and to determine whether mind–body exercise improves the connectivity of the brain’s nerve fibers in patients with MCI. Functional near-infrared spectroscopy can monitor local hemodynamic changes in the brain, revealing, in real time, the relationship between mind–body exercise and the pathological process of dementia. Monitor local hemodynamic changes in the brain, revealing changes in brain activation areas during mind–body exercise.^[55]^ Molecular markers like β-amyloid and tau protein also play a key role in the diagnosis and monitoring of dementia. Detecting changes in biomarker levels, such as β-amyloid and tau protein, can help clarify whether mind–body exercise can intervene in the pathological process of dementia, such as reducing amyloid deposition and inhibiting the abnormal phosphorylation of tau protein, thereby providing more direct evidence for the neurobiological mechanisms of mind–body exercise. Provide more direct evidence for the neurobiological mechanisms of mind–body exercise.^[56]^ The potential interventional effects of mind–body exercise on the pathological process of dementia can be better understood. This integrated research approach could help develop new therapeutic strategies and provide strong support for clinical trials.^[57]^

Although the current study focused on single-mode repetitive aerobic exercise (which did not demonstrate significant cognitive benefits for older adults with MCI) other forms of aerobic activity have been shown to mitigate age-related brain atrophy and slow cognitive decline.^[27,57–59]^ Prior research indicates that while standalone aerobic exercise provides modest cognitive improvements, its effects may be constrained compared to integrated interventions. Specifically, combined approaches that pair mind–body exercises with aerobic training and cognitive stimulation have yielded superior outcomes in enhancing cognitive function among older adults with MCI.^[60]^ These findings suggest that the design of aerobic exercise protocols (such as incorporating varied movement patterns or cognitive engagement) alongside multimodal interventions, may be critical to optimizing therapeutic effects. By transcending the limitations of single-type repetitive aerobic models, comprehensive programs that address both physiological and neurocognitive pathways could offer more robust strategies for managing cognitive decline in aging populations.

Future research could explore multimodal exercise interventions that integrate structured aerobic routines with mind–body practices to leverage the complementary benefits of both approaches. By incorporating mind–body exercise components (such as cognitive–motor tasks or breath-regulation techniques) into existing aerobic training frameworks, these combined programs can synergize the cardiovascular and metabolic advantages of aerobic exercise with the neurocognitive stimulation provided by mind–body modalities. Empirical investigations should focus on assessing the impact of these integrated interventions on cognitive function in older adults with MCI, while systematically optimizing parameters such as exercise intensity, session duration, and the ratio of aerobic to mind–body components. Through iterative adjustments to these intervention characteristics, researchers can identify the most effective multimodal protocols that enhance cognitive outcomes by addressing both physiological health and higher-order neural processes. This approach holds promise for developing tailored exercise programs that maximize therapeutic benefits by capitalizing on the unique strengths of each modality within a unified intervention framework.

A limited number of studies were included in this study, and some of the findings were highly heterogeneous. Most of the included mind–body exercise literature was from Asia, and the study populations differed geographically and culturally. The inclusion of outcome indicators is limited and may not fully reflect improvements in cognitive function. In the future, we can continue to further clarify the effects of mind–body exercise and structured aerobic exercise on cognitive function in elderly people with MCI by conducting multi-center, large-sample, and high-quality studies, which expected to provide more effective exercise intervention strategies for the prevention and treatment of MCI, promote the development of this field, and make greater contributions to the improvement of cognitive health in the elderly.

## 5. Conclusion

Analysis of exercise interventions for older adults with MCI suggests that mind–body exercise may be more beneficial than structured aerobic exercise for improving cognitive function. However, these findings should be interpreted with caution due to heterogeneity in some analyses, the limited number of included studies, and geographical bias (predominantly Asian populations). Further high-quality, multi-center trials are needed to confirm these preliminary results and establish optimal intervention protocols. Based on current evidence, mind–body exercise appears to be a promising non-pharmacological approach for supporting cognitive health in MCI and warrants prioritization in clinical practice where appropriate.

## Author contributions

**Conceptualization:** Jie Chen, Chenggen Peng.

**Data curation:** Yuheng Yin, Songlin Liu.

**Formal analysis:** Yuheng Yin, Ziyi Chen, Shan Chen.

**Methodology:** Xiang Bo Wang, Chenggen Peng.

**Resources:** Linxiao Xiao.

**Software:** Xiang Bo Wang, Hui Huang.

**Validation:** Songlin Liu.

**Writing – original draft:** Xiang Bo Wang, Yingli Liu.

**Writing – review & editing:** Xiang Bo Wang, Chenggen Peng.

## Supplementary Material


