# Supplementary material for: The effects of structured aerobic exercise and mind–body exercise on cognitive function in older adults with MCI: Systematic review and meta-analysis
Source: Medicine (Baltimore). 2026 Mar 6;105(10):e47633. doi: 10.1097/MD.0000000000047633 (PMC12975272; doi:10.1097/MD.0000000000047633)

**Figure S1:** Funnel plot of standard error vs. effect size for publication bias assessment.


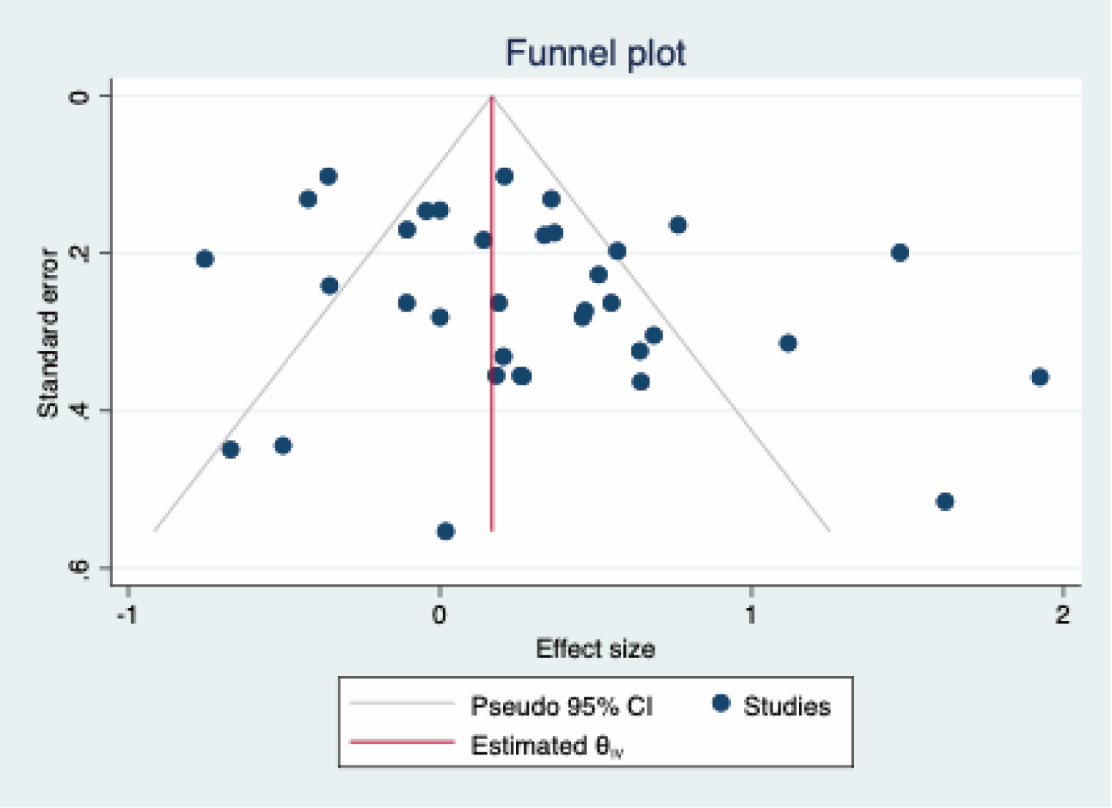


**Figure S2:** Egger’s regression plot of SND of effect estimate vs. precision for publication bias evaluation.


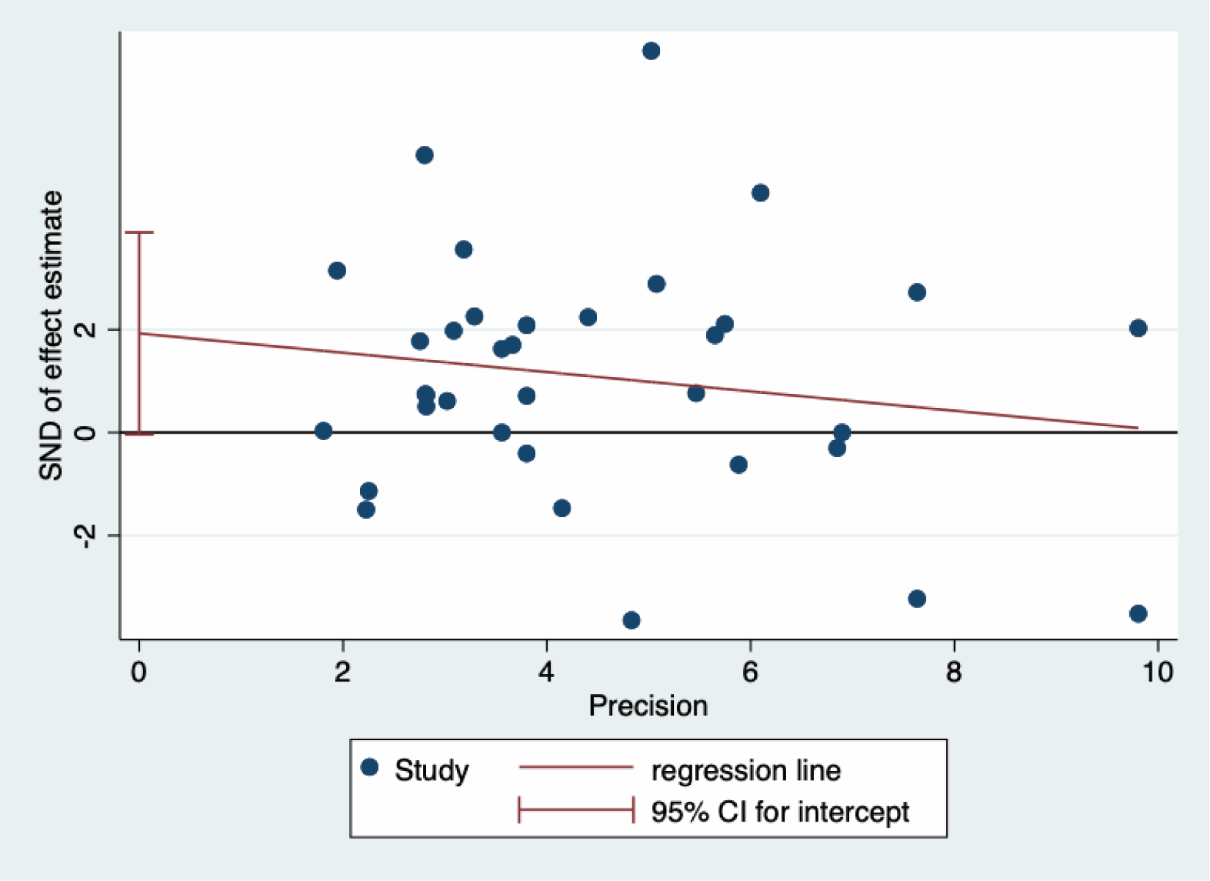


**Figure S3:** Effect sizes of forest plots for structured aerobic exercise versus controls—MMSE outcomes. MMSE, Mini-Mental State Examination.


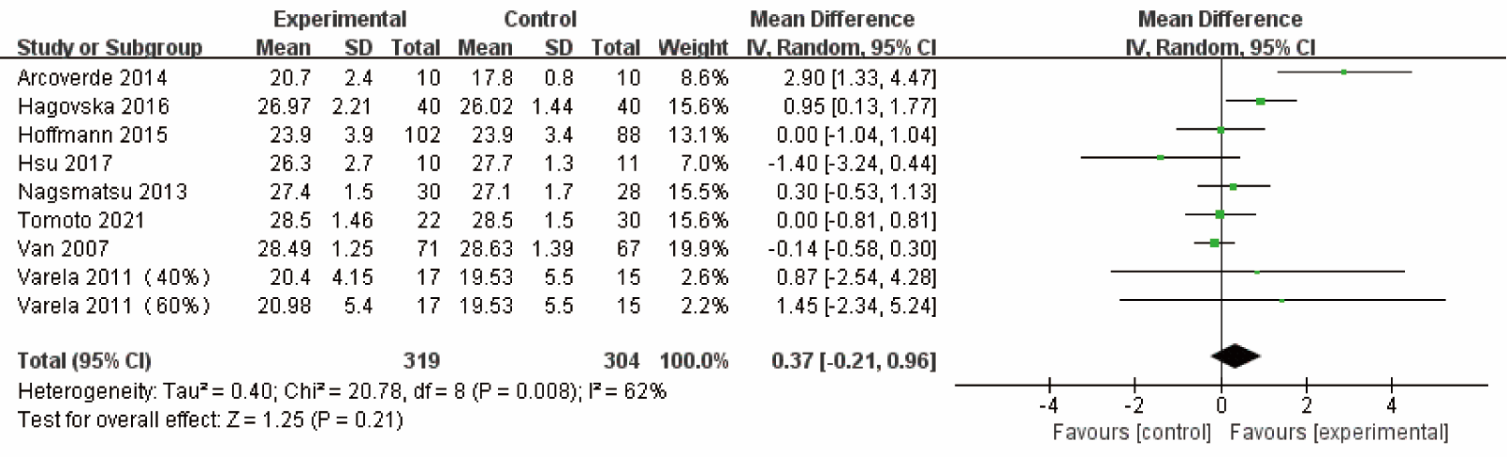


**Figure S4:** Effect sizes of forest plots for mind-body exercise versus controls—MMSE outcome. MMSE, Mini-Mental State Examination.


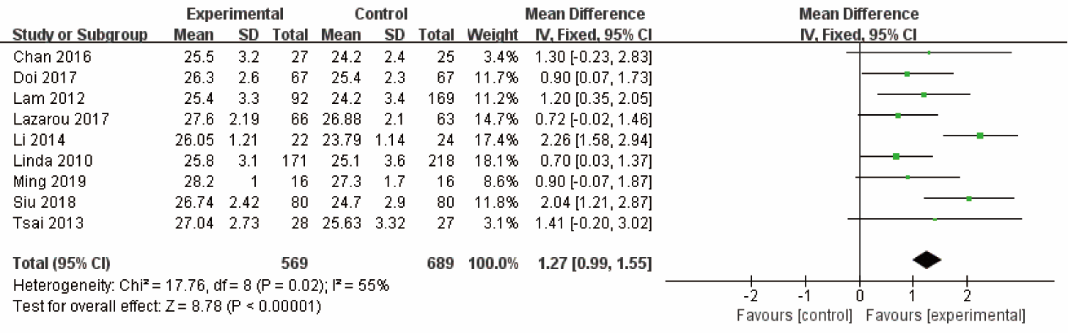


**Figure S5:** Effect sizes of forest plots for mind-body exercise versus controls—MOCA outcomes. MoCA, Montreal Cognitive Assessment.


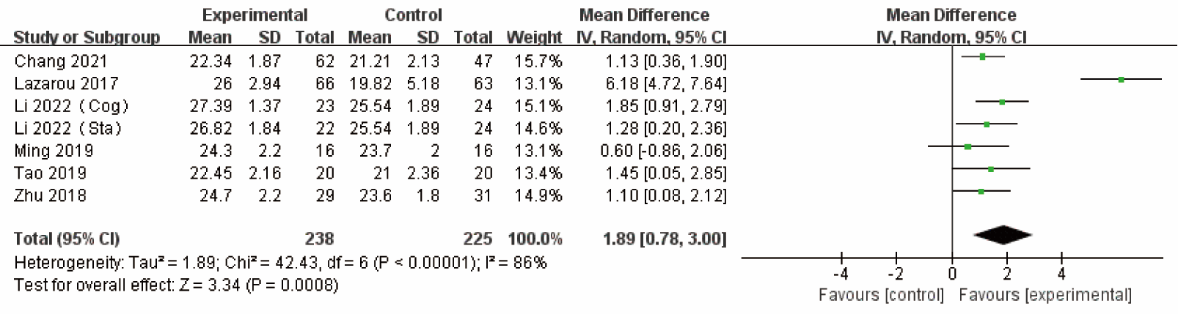


**Figure S6:** Effect sizes of forest plots for structured aerobic exercise versus controls—ADAS-Cog outcomes. ADAS-Cog, Alzheimer’s Disease Assessment Scalee-Cognitive Subscale.


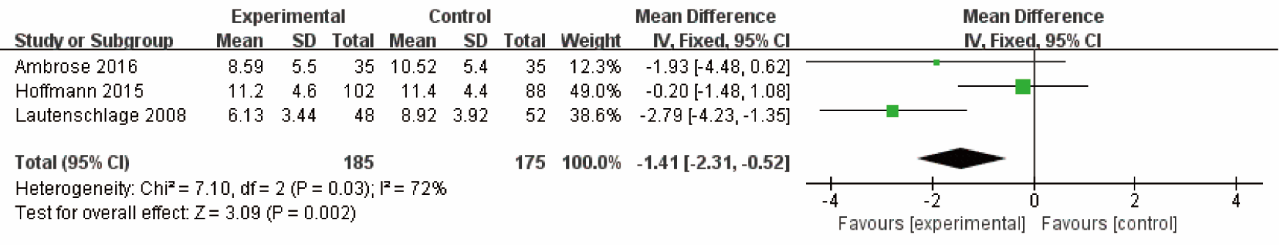

Supplement: Supplementary file 1 [file medi-105-e47633-s001.docx]
